# High treatment resistance is associated with lower performance in the Stroop test in patients with obsessive–compulsive disorder

**DOI:** 10.3389/fpsyt.2023.1017206

**Published:** 2023-05-05

**Authors:** Damien Doolub, Nicolas Vibert, Fabiano Botta, Ali Razmkon, Cédric Bouquet, Issa Wassouf, Bruno Millet, Ghina Harika-Germaneau, Nematollah Jaafari

**Affiliations:** ^1^Centre de Recherches sur la Cognition et l’Apprentissage, CNRS UMR 7295, Université de Poitiers, Poitiers, France; ^2^Centre de Recherches sur la Cognition et l’Apprentissage, CNRS UMR 7295, Université de Tours, Tours, France; ^3^Unité de Recherche Clinique Pierre Deniker du Centre Hospitalier Henri Laborit, Poitiers, France; ^4^Research Center for Neuromodulation and Pain, Shiraz, Iran; ^5^Centre Hospitalier du Nord Deux-Sèvres, Service de Psychiatrie Adulte, Thouars, France; ^6^Institut du Cerveau et de la Moelle, UMR 7225 CNRS, INSERM, Sorbonne Université et Département de Psychiatrie Adulte, Groupe Hospitalier Pitié-Salpêtrière, Paris, France

**Keywords:** obsessive–compulsive disorder, executive functions, treatment resistance, insight, working memory, inhibition of prepotent responses, task-switching

## Abstract

Around 50% of the patients with obsessive–compulsive disorder (OCD) are resistant to treatment, and patients with OCD show alterations in a broad range of cognitive abilities. The present study investigated the links between treatment-resistance, executive and working memory abilities, and the severity of OCD symptoms among 66 patients with OCD. The patients performed seven tests gauging their executive functions and working memory and filled in questionnaires for OCD severity and insight into their pathology. In addition, the executive and working memory abilities of a subset of these patients were compared with those of individually matched control participants. In contrast with previous studies, patients’ treatment resistance was evaluated by considering the clinical outcomes of all the treatments that they received during the course of their disease. Higher treatment resistance was associated with lower performance in one particular executive test, the Stroop test, which assessed patients’ ability to inhibit prepotent/automatic responses. Older age and more severe OCD symptoms were also associated with higher treatment resistance. Regardless of OCD severity, the patients displayed small to moderate deficits across most components of executive functions compared to control participants. Interestingly, patients with OCD took more time than control participants to perform speeded neuropsychological tests but never made more errors. Altogether, this study shows that the treatment-resistance of patients with OCD may be reliably quantified over the course of years and treatments using Pallanti and Quercioli’s (2006) treatment resistance-related scales. The data suggest that the Stroop test could be used clinically to anticipate treatment outcomes in to-be-treated patients.

## 1. Introduction

Obsessive–compulsive disorder (OCD) is challenging to treat as in about half of the patients, many symptoms persist even after several treatment trials (1, 2). The patients’ executive abilities and insight into the pathology have been linked to treatment resistance (3–5), but no consensus exists about the exact nature and strength of these relationships (6, 7). These divergences are mainly due to an absence of unanimity in defining treatment resistance across previous studies and to the diversity of tools that were used to assess executive functions (8–10).

Executive functions are a set of high-level cognitive processes that combine different general-purpose control functions, which select and monitor behaviors to attain chosen goals. They allow individuals to shift their mindsets and reorient their cognitive resources depending on the ongoing tasks while inhibiting inappropriate behavioral responses and rejecting irrelevant material (11, 12). OCD patients exhibit broad deficits of moderate effect size across nearly all domains of executive functions and attentional control (13–16). Miyake and Friedman (11, 17) have proposed the most widely accepted cognitive model of executive functions (18, 19), which can be used as a framework to assess the relationships between executive functions and OCD patients’ clinical features.

The present study investigated the links between treatment-resistance, executive abilities, insight, and OCD severity among 66 patients. In addition, the executive and working memory abilities of 36 patients were compared with those of 36 control participants who were individually matched with patients for age and educational level. In the present experiment, in contrast with most previous studies, the tests used to assess executive functions precisely targeted one or several of the core executive functions components defined by Miyake and Friedman (17). Indeed, as stated by Kashyap and Abramovitch (14), carefully selecting tests to assess specific domains of cognitive function should be standard practice in neuropsychological research. Another originality of the study was that the level of treatment resistance of the patients was evaluated by considering the clinical outcomes of all the treatments they have received since the onset of OCD symptoms and until the day they were tested in this study.

Miyake and Friedman’s model (17) relies on three weakly intertwined fundamental components: *inhibition, set-shifting,* and *working memory updating*. The *inhibition* component includes two distinct sub-components: inhibition of prepotent, automatic responses, and resistance to interference from distracting information (attentional control; (20, 21). *Set shifting* is “the disengagement of an irrelevant task set and the subsequent active engagement of a relevant task set” (22) and is linked to cognitive flexibility, task-switching, and supervision. Finally, *updating* entails constant monitoring and rapid addition/deletion of working memory contents (17). The efficiency of access to long-term memory, i.e., verbal fluency (16, 23), may also depend on *updating* abilities because it involves transferring information from long-term memory to working memory (17).

In relation to the three components of Miyake and Friedman’s model, OCD patients display small to medium alterations of inhibition of prepotent responses, as assessed by the Stroop test, and moderate deficits in task-switching and attentional set-shifting (15, 16, 24, 25). According to Snyder et al. (16), OCD patients would exhibit more significant deficits in the updating component of executive functions, coherent with their incapacity to disengage themselves from their obsessions and compulsions. In addition, they display slight deficits in verbal working memory and more substantial deficits in visuospatial working memory (16, 26, 27). Finally, OCD moderately impacts verbal fluency tests (16, 27).

Known predictors of remission in OCD are early diagnosis and initiation of treatment, shorter duration of untreated OCD, and less severe OCD symptoms (1, 5, 28, 29). Conversely, a slow and steady evolution of OCD, being over 40 years of age, and the number of admissions in psychiatry would be predictors of treatment resistance (1, 29). In addition, the patients who experience only partial remission after first-line treatment with selective serotonin reuptake inhibitors (SSRI) antidepressants and/or cognitive behavioral therapy (CBT) have a much higher relapse rate (1, 29).

Regarding the links between treatment-resistance and executive functions, a few studies reported that patients showing better executive performance were more likely to respond to CBT and fluoxetine (4, 30), but other ones failed to show any difference in executive functioning between CBT-responsive and CBT-resistant OCD (31–33). Two studies found that patients who made more perseverative errors in tests responded *better* to fluoxetine (4, 34). However, most of these studies only assessed the patients’ treatment resistance to only one treatment or set of treatments over a relatively brief period. They did not consider all the other treatments the patients had received during the course of their disease. Therefore, no firm conclusion can be drawn about the impact of executive abilities on treatment resistance. Finally, OCD patients with poor insight would display more resistance to treatments (3, 5, 28, 35) and more significant executive deficits (36) than patients with better insight. Nevertheless, conflicting data were obtained since some authors did not report any relationship between insight and response to CBT or serotonin reuptake inhibitors (1, 37).

Altogether, higher treatment resistance was expected to be associated with lower performance on some executive and/or working memory tests (hypothesis 1) and lower insight (hypothesis 2). A third hypothesis was that patients with OCD should show broad alterations in executive and working memory abilities compared to healthy controls but may exhibit more significant deficits in the updating component of executive functions (16).

## 2. Materials and methods

### 2.1. Participants

Sixty-six consecutive outpatients (65 adults and one 16-year-old teenager) with a primary diagnosis of OCD were recruited at a specialized psychiatric hospital within a period of 4 years after clinical examination by well-trained psychiatrists. A preliminary power analysis was conducted to estimate the number of participants to include in the multiple regressions used to test hypotheses 1 and 2, assuming an expected large effect size of 0.50 (Cohen’s *f^2^* for multiple regression), a power of 0.80, and a significance level set at 0.05, and yielded a minimum sample size of 47 patients. The Mini International Neuropsychiatric Interview (MINI version 5.0.0) was used to affirm the presence of obsessive–compulsive symptoms according to DSM-IV TR criteria and to assess other psychiatric comorbidities (38). At inclusion, all patients had a score of 16 or greater on the French version of the Yale-Brown Obsessive–Compulsive Scale (Y-BOCS), which measures the severity of OCD symptoms on a 0 to 40 (maximum severity) scale (39). Participants with current or former severe or decompensated mood disorders, schizophrenia, and/or addictive disorders (except tobacco smoking) were excluded.

At the time of inclusion and testing, 65 patients, including all patients who were compared with matched controls, were under medication as detailed in Table 1.

**Table 1:** Summary of patients’ treatments at inclusion.

	All OCD patients (*n* = 66)	OCD patients with matched controls (*n* = 36)
**No treatment**	**1**	**0**
**Drug treatment**	**65**	**36**
SSRI	9	4
SSRI + antipsychotics	2	1
SSRI + anxiolytics	4	2
SSRI + other classes of antidepressants	2	1
SSRI + antipsychotics and anxiolytics	4	2
SSRI + antipsychotics + anxiolytics + other classes of antidepressants	18	9
SSRI + antipsychotics + anxiolytics + other classes of antidepressants + mood stabilizers	12	8
SSRI + other classes of antidepressants + antipsychotics	1	0
SSRI + other classes of antidepressants + anxiolytics	6	5
SSRI + other classes of antidepressants + anxiolytics + mood stabilizers	2	2
Other classes of antidepressants	2	2
Other classes of antidepressants + antipsychotics + anxiolytics	2	0
Other classes of antidepressants + antipsychotics + mood stabilizers	1	0
**At least one non-drug therapy**	**63**	**33**
CBT or other type of psychotherapy	22	7
CBT or other type of psychotherapy + rTMS	16	11
CBT or other type of psychotherapy + tDCS	5	3
CBT or other type of psychotherapy + rTMS + tDCS	14	7
Deep brain stimulation (DBS)	1	1
DBS + CBT	2	1
DBS + CBT + rTMS	2	2
DBS + CBT + tDCS	1	1

SSRI: selective serotonin reuptake inhibitors, CBT: cognitive behavioral therapy, rTMS: repetitive transcranial magnetic stimulation, tDCS: transcranial direct current stimulation, DBS: deep brain stimulation.

The cognitive abilities of a subset of 36 patients were compared with those of control participants who were individually matched with patients by sex, age (to within 5 years), and educational level (Table 2). The prior power analysis that was conducted to estimate the number of patients and control participants to include in the comparison, assuming an expected medium effect size of 0.50 [Cohen’s *d*, see (40)], a power of 0.80, and a significance level set at 0.05, yielded a minimum sample size of 27. Controls were recruited from the local community and had no history of current psychiatric or neurological illness, as assessed by the MINI, clinical examination, and the medical files. All participants consented to participate in the study, and the local ethics committee approved the experimental protocol.^1^

**Table 2:** Mean values ± standard deviations (SD) of participants’ characteristics and patients’ clinical variables at the time of testing (except otherwise stated).

	All OCD patients (*n* = 66)	OCD patients with matched controls (*n* = 36)	Healthy controls (*n* = 36)
Age (yrs)	38.3 ± 12.9 (range 16 to 73)	37.1 ± 12.7 (range 16 to 73)	37.0 ± 12.3 (range 15 to 68)
Gender (F/M)	37/29	20/16	20/16
Age at onset of pathology (yrs)	16.7 ± 11.2 (range 3 to 64)	15.9 ± 10.2 (range 3 to 64)	N/A
Duration of the disease (yrs)	21.2 ± 12.6 (range 2 to 58)	20.8 ± 10.9 (range 2 to 58)	N/A
Age at diagnosis (yrs)	28.6 ± 12.8 (range 6 to 69)	28.9 ± 10.8 (range 14 to 69)	N/A
Duration of OCD treatment (yrs)	10.1 ± 9.6 (range 0 to 36)	8.2 ± 7.7 (range 0 to 27)	N/A
Educational level (yrs)	12.3 ± 2.6	12.9 ± 2.4	12.9 ± 2.4
HAM-A score	13.8 ± 5.5 (range 7 to 29)	13.0 ± 4.6 (range 8 to 29)	N/A
Y-BOCS score	27.3 ± 4.8 (range 17 to 37)	26.4 ± 4.4 (range 17 to 34)	N/A
BABS score (insight)	7.8 ± 4.5 (range 1 to 18)	7.3 ± 4.0 (range 3 to 18)	N/A
Pharmacological treatment	65 patients	36 patients	None
Current comorbidity	50 patients	30 patients	None
Anorexia nervosa	6 patients	3 patients	None
Bipolarity	1 patient	1 patient	None
Generalized anxiety disorder	12 patients	5 patients	None
Mild depressive episode	14 patients	8 patients	None
Obsessive compulsive personality disorder	7 patients	5 patients	None
Sleep disorders	21 patients	12 patients	None
Social phobia	4 patients	1 patient	None
Tics	6 patients	2 patients	None

N/A: not applicable.

The Department of Medical Information of the psychiatric hospital approved access to the patients’ medical files in paper and/or electronic format. Each file was anonymized, and the data collected were classified in an Excel® workbook. The medical data, including the types of obsessions and compulsions displayed by the patient, were listed according to the clinical observations and the checklist of the Y-BOCS. The retrospective treatment inventory that was used to assess the patients’ treatment resistance included all the treatments patients received over the course of their disease and their respective clinical outcomes.

### 2.2. Assessment of OCD patients’ treatment resistance

Treatment resistance was characterized and assessed when the executive and/or working memory tests were performed, considering retrospectively all the psychotropic drugs and psychological treatments the patients received from the beginning of obsessive–compulsive symptoms until the testing time (Table 2). We used adapted versions of the two scales of treatment resistance designed by Pallanti and Quercioli (2), i.e., the “levels of non-response to treatments” (LNRT, Table 3) and “stages of response to treatments” (SRT, Table 4) scales. In both cases, large numbers reflect higher treatment resistance. The sample of patients was heterogeneous and included both monotherapy-treated and polytherapy-treated patients, with a range of possible outcomes from aggravated illness to complete remission and with either an episodic or a continuous course of the disease.

**Table 3:** Adaptation of Pallanti and Quercioli’s (2) scale of levels of non-response to treatment (LNRT scale).

LNRT	Description of the corresponding treatment
I	SSRI or CBT and/or psychotherapy
II	SSRI plus CBT and/or psychotherapy
III	2 SSRIs tried plus CBT and/or psychotherapy
IV	At least 3 SSRIs tried plus CBT and/or psychotherapy
V	At least 3 SRIs (including Clomipramine) tried plus CBT and/or psychotherapy
VI	At least 3 SRIs tried (including Clomipramine augmentation) plus CBT and/or psychotherapy
VII	At least 3 SRIs (including Clomipramine) tried with or without CBT and/or psychotherapy, plus psychoeducation and other classes of drugs (benzodiazepine, mood stabilizer, neuroleptic, psychostimulant)
VIII	At least 3 SRIs (including intravenous Clomipramine) tried with or without CBT and/or psychotherapy plus psychoeducation
IX	At least 3 SRIs (including Clomipramine) tried with or without CBT and/or psychotherapy, plus psychoeducation and other classes of antidepressants (MAOIs, NSRI)
X	All above treatments + neurosurgery or deep brain stimulation

NSRI: norepinephrine-serotonin reuptake inhibitor.

**Table 4:** Adaptation of Pallanti and Quercioli’s (2) scale of stages of response to treatment (SRT scale).

Stage of response		Treatment outcome description
I	Recovery	Y-BOCS score < 8 or perception of complete recovery by the patient and physician, not at all ill
II	Remission	Y-BOCS score < 16, or marked improvement repeatedly perceived by the patient and physician
III	Full response	35% or greater reduction of the Y-BOCS score, or marked improvement in the symptoms perceived by the patient and physician, with some residual symptoms
IV	Partial response	25 to 35% reduction of the Y-BOCS score, or moderate decrease in the symptoms perceived by the patient and physician
V	Non-response	Less than 25% reduction of the Y-BOCS score, no improvement with dose adjustments and/or changes of therapy
VI	Relapse	25% or greater increase of Y-BOCS score and symptom recurrence following remission or partial or full response after at least 3 months of treatment
VII	Refractory	No change or worsening with all available therapies

#### 2.2.1. Scales of treatment resistance

##### 2.2.1.1. Levels of non-response to treatments.

The LNRT scale (2) includes 10 levels of successive treatments that ought to be prescribed to OCD patients up until clinical response is observed. Indeed, the staging of successive treatments for OCD (41–43) begins with first-line treatments such as selective serotonin reuptake inhibitors (SSRI) and/or cognitive and behavioral therapy (CBT). If no satisfactory outcome is obtained, a switch to another SSRI or clomipramine or augmentation strategies such as adding serotonin and norepinephrine reuptake inhibitors (SNRI), atypical antipsychotics, and/or intravenous injections of clomipramine are considered. Finally, several neurosurgery techniques are effective for treatment-resistant patients and are used for the most severe cases (44, 45).

The original LNRT scale (2) had to be slightly adapted (Table 3) to consider the local specificities in care management. Indeed, first-line drug treatments were not always associated with CBT, as only 46 patients had CBT, but were associated with other types of evidence-based psychotherapy (46), including psychodynamic psychotherapies for six patients. Hence, the LNRT scale was modified by changing “CBT” to “CBT and/or psychotherapy.” Another difference with the original scale was that whatever the stage of treatment they reached, all OCD patients could be treated with focal rTMS and/or tDCS targeting various OCD-related brain areas. Otherwise, the staging of the drug treatments was the same as that described in the original scale.

##### 2.2.1.2. Stages of response to treatments

The SRT scale is the second scale that Pallanti and Quercioli (2) designed to assess treatment-resistance in OCD. The scale classifies treatment outcomes into seven levels based on the evolution of the patients’ Y-BOCS scores and/or scores in the Clinical Global Impressions (CGI) scale (47). Our adaptation of the SRT scale (Table 4) considered the evolution of the patients’ Y-BOCS scores as well as the subjective perception of improvements in everyday life by the patient and his / her psychiatrist, as recommended by Mataix-Cols et al. (48), but did not consider the CGI scale, which was not used in local clinical practice. Otherwise, the Y-BOCS cut-off scores for recovery and remission, and the thresholds for the percentages of reduction or increase of the Y-BOCS score used to define the different levels of response, were the same as in the original scale [see (2), for details]. The different scores of the Y-BOCS retained in the present study were those obtained during each patient’s medical follow-ups and at the time of testing. Percentages of reduction or increase in Y-BOCS scores were calculated considering a period of at least 4 weeks between two Y-BOCS submissions. Generally, Y-BOCS scores were obtained on the first day of treatment and after eight and then 12 weeks or more of treatment (end of the protocol). For a few patients who were already treated for OCD at the beginning of the 2000s, no Y-BOCS scores were available. What was used was only the subjective percentage of improvement or worsening perceived by the patient and his / her psychiatrist after several weeks of treatment. In line with current international guidelines for treating OCD (44, 49), other criteria included decreasing or increasing the time devoted to obsessions and/or compulsions and whether the treatment was modified or discontinued after several months.

### 2.3. Experimental procedure

The cognitive abilities of patients with OCD were assessed by using tests targeting precisely one or several of the core executive components defined by Miyake and Friedman (17). Most of the participants (all 36 healthy controls and 53 out of the 66 patients) performed seven tests assessing their working memory (2 tests) and executive abilities (5 tests), always in the same order. Ten patients who were enrolled at the beginning of the study were only asked to perform the two working memory tests. The other participants began with three pen-and-paper tests: a verbal, semantic fluency test that taps the updating component of executive functions but also depends on language abilities, Golden’s (50) version of the Stroop test (inhibition of automatic/prepotent responses), and the d2 sustained attention test (resistance to interference from distracting information). The other tests were computerized and included the reading span test (verbal working memory), the Hayling sentence completion test (updating component and inhibition of prepotent responses), Monsell and Mizon’s (51) task-switching test (set shifting component), and the backward location span test (visuospatial working memory), in that order.

Pre-inclusion was done within 1 week before undertaking the neuropsychological tasks. Participants were administered French versions of the Hamilton Anxiety Rating Scale [HAM-A, (52)], which measures anxiety level on a 0 to 56 (maximum severity) scale, and of the Brown Assessment of Beliefs Scale [BABS, (53)]. The BABS assesses patients’ insight by scoring from 0 (highest insight) to 24 (lowest insight).

### 2.4. Cognitive tests

#### 2.4.1. Tests of executive functions

A stopwatch was used to time the pen-and-paper tests. For the Hayling test and the task-switching test, E-Prime® 2.0 software was used to control the presentation of stimuli, timing operations, and data collection. Response times were measured using a voice key for the Hayling test and a serial response box for the task-switching test.

##### 2.4.1.1. Verbal fluency test

Within 60 s, participants must cite a maximum of exemplars of the “animals” category. The score is the number of different animal names given by the participant.

##### 2.4.1.2. Stroop test

A paper version of the Stroop test (50, 54) was used. On the first card (card A, word-reading condition), participants must read as many as possible color names (red, green, blue, or yellow) written in black ink within 45 s. On card B (color-naming condition), participants must give the color of as many as possible groups of Xs printed in color within 45 s. Finally, on card C (color-word condition), participants must give the ink color of as many as possible color names printed with a color different from that indicated by their name (for example, the word RED in blue font) within 45 s, which requires inhibition of the prepotent tendency to read the color word. Participants’ scores on cards A, B, and C were used to calculate two distinct inhibition-related scores (55, 56). The first one was the difference between the scores obtained in the color-naming and color-word conditions. The smaller the difference, the higher the ability to inhibit the prepotent tendency to read the color words on card C. The second one was Golden’s inhibition score, calculated in two steps. Firstly, the participant’s scores on cards A and B are used to predict their score on card C according to the formula P = (A × B)/(A + B). Then, the prepotent inhibition score (PI) is calculated by subtracting the predicted score P from the participant’s real score on card C (PI = C − P). Altogether, positive and negative Golden’s inhibition scores indicate higher or lower than average inhibition abilities, respectively.

##### 2.4.1.3. d2 attention control test

The d2 test of attention control (57) consists of 14 successive lines, each containing 47 characters. All characters are either “p” or “d” with one to three dashes presented above and / or below each character. Participants have 20 s to scan each line from left to right to detect and mark all “d” characters with exactly two dashes as quickly and accurately as possible. Participants’ performance is evaluated by counting the number of characters that were processed (GZ) and the number of omission and commission errors (F), which were used to compute participants’ error rates (F/GZ, in %) and final test scores (GZ minus F).

##### 2.4.1.4. Hayling sentence completion test

In Burgess and Shallice’s (58) version of the task, participants must complete a series of 32 sentences from which the last word is omitted. The task involves two conditions. In the completion condition, participants are required to give a word that completes the sentence in a meaningful way as quickly as possible. Response times indicate participants’ ability to recover relevant items in long-term memory, tapping the updating component of executive functions. In the inhibition condition, participants must, in contrast, suppress obvious responses and produce a word unrelated to the sentence. In that condition, the response times and error rates indicate the participants’ ability to inhibit prepotent responses. Each of the 32 sentences is randomly assigned to either the completion or the inhibition condition, providing that 16 are assigned to each condition.

##### 2.4.1.5. Task-switching test

Monsell and Mizon’s (51) task-switching test taps both the set-shifting and resistance to interference components of executive functions. The target stimuli include congruent, incongruent and neutral stimuli. They are based on a horizontal arrow that can point either to the left or to the right, and with either the word “LEFT” or “RIGHT” written in the center of the shaft. Participants are instructed to press as fast as possible, but without making mistakes, either the rightmost or the leftmost button of the response box according to the word indicated within the arrow when the arrow appears within the top half of the screen (the “word task”), and according to the direction indicated by the arrow when the arrow appears at the bottom half of the screen (the “arrow task”). In congruent stimuli, the arrowhead and the word indicate the same direction, whereas, in incongruent stimuli, the arrowhead and the word indicate opposite directions. The neutral stimuli are control stimuli where either the direction arrow has a string of five Xs inside or the word indicating the direction is written in a rectangle corresponding to the arrow shaft alone.

The association between the stimulus location and the task to perform is counterbalanced between participants. Participants perform four blocks of 36 trials, and the program ensures that participants perform each of the two tasks on 24 congruent, 24 incongruent, and 24 neutral stimuli. In addition, half of the trials are “task-repetition trials” where participants must perform the same task as in the previous trial, whereas the other trials are “task-switch trials,” where the task to perform changes compared to the previous trial. Again, participants perform repetition and switch trials on 24 congruent, 24 incongruent, and 24 neutral stimuli.

Participants’ response times are collected on each trial and averaged according to the nature of the stimuli and whether they are task-repetition or task-switch trials. Participants’ set-shifting ability is evaluated by measuring the task-switching cost, i.e., the difference between reaction times to task-switch trials and to task-repetition trials. Participants’ ability to resist interference is evaluated by measuring the incongruency cost, i.e., the difference between reaction times to incongruent trials and to congruent trials.

#### 2.4.2. Working memory tests

##### 2.4.2.1. Reading span test

A French version of the test (59) was used to evaluate participants’ ability to maintain and update information in verbal working memory. The material consists of 100 unrelated test sentences. Participants are instructed to read aloud at their own pace sets of two to six unrelated sentences, which are presented one by one on the computer screen, and to memorize the final word of each set sentence. After participants have read all sentences in a set, they must restitute the last word of each sentence. The experimental session includes five successive blocks of five sets of sentences, i.e., one two-sentence, one three-sentence, one four-sentence, one five-sentence, and one six-sentence set. The score is the total number of correctly recalled final words (60).

##### 2.4.2.2. Backward location span test

The backward location span task (61) is used to evaluate participants’ ability to maintain and update information in visuo-spatial working memory. In each trial, participants are shown a five-by-five grid in which a sequence of randomly located cells turns black one after the other. After seeing each sequence, participants are requested to reproduce it in the opposite order by clicking on the corresponding cells in an empty grid. The experimental session includes 32 sequences of increasing difficulty, namely four two-cell sequences, four three-cell sequences, and so on, up to four nine-cell sequences. The participant’s visuospatial span score is the total number of correctly recalled cells, with a maximum possible score of 176.

### 2.5. Data analysis

All statistical analyses were performed with Statistica® 14.0.0.15 software, with the significance threshold set at 5%. A descriptive analysis of patients’ treatment resistance scores was conducted as a first step. To identify groups of patients showing similar resistance profiles when considering both resistance scores together, the patients’ LNRT and SRT scores were entered in a cluster analysis based on the *k*-means method. The main goal of this analysis was to identify a subset of highly resistant patients who had both high LNRT scores and high SRT scores, and a subset of non-resistant patients who had both low LNRT scores and low SRT scores, while discarding the patients displaying contrasted resistance scores (i.e., either low LNRT scores and high SRT scores or high LNRT scores and low SRT scores). Hence, the number of clusters to define was set in advance at 4.

Descriptive correlational analyses were then conducted to assess the links between the main demographic and clinical variables of patients with OCD (age, gender, Y-BOCS scores, insight scores and anxiety level) and between these variables and the patients’ scores on executive and working memory tests. Correction for multiple testing was performed using the procedure described by Benjamini and Hochberg (62).

The raw correlations between the patients’ scores on executive and working memory tests and their treatment-resistance scores were assessed. However, because testing numerous correlations increases the risk of erroneously finding significant correlations, hypotheses 1 and 2 were tested by designing multivariate regression models to assess whether the patients’ treatment resistance scores could be predicted by their scores on cognitive tests and/or clinical variables. In addition, to get a complete picture of what determines patients’ treatment resistance, hypotheses 1 and 2 were also tested by comparing (using t-tests for independent samples) the clinical variables and the scores on cognitive tests of the highly resistant versus non-resistant patients identified by the cluster analysis.

Finally, to test hypothesis 3, Student’s *t*-tests for paired samples were performed to compare the executive and working memory abilities of 36 patients with OCD with those of their matched controls. Since the patients were expected to show lower cognitive abilities than healthy controls, one-tailed *t*-tests were used. Effect sizes were evaluated with Cohen’s *d*. Again, correction for multiple comparisons was performed using Benjamini and Hochberg’s (62) procedure.

## 3. Results

### 3.1. Treatment resistance in patients with OCD

The treatment-resistance of one patient could not be determined because of missing data in the medical file. The resistance scores of the 65 remaining patients were highly heterogeneous. The average LNRT score was *M* ± SD = 6.0 ± 3.3, but the individual scores covered the complete range of the scale, from 1 to 10. The average SRT score was 4.8 ± 2.0, with individual scores covering the complete range of the scale, from 1 to 7. Patients’ LNRT and SRT scores were positively correlated (*r* = 0.52, *p* < 0.001) and did not significantly depend on patients’ gender (LNRT *t* = 1.62, *p* = 0.11, SRT *t* = 1.63, *p* = 0.11).

**Figure 1:**
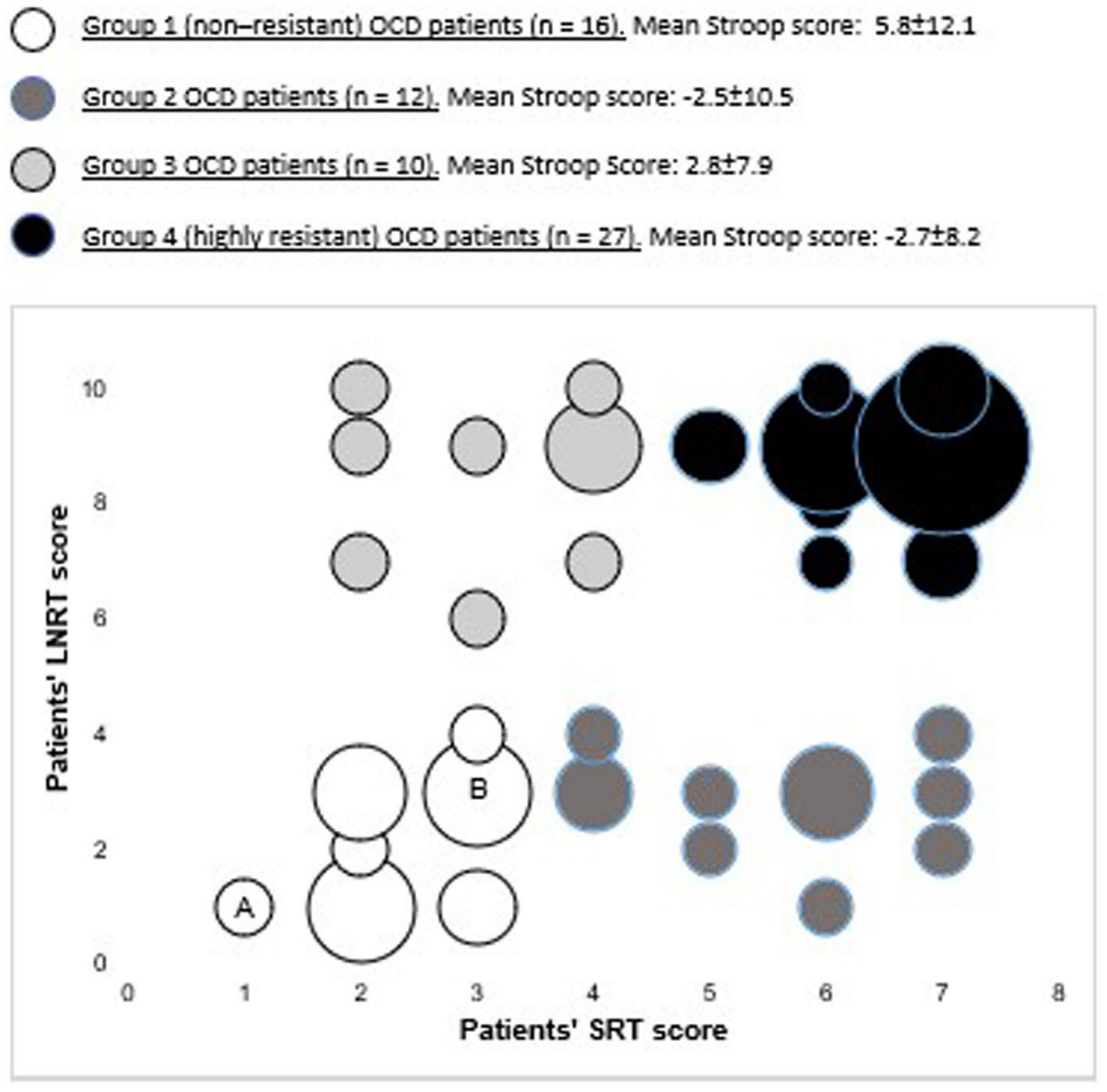
Graph showing the treatment resistance scores of the four groups of patients with OCD identified according to their resistance profiles by the cluster analysis of patients’ LNRT and SRT scires. The size of the circles reflects the number of patients who had the same particular combination of LNRT and SRT scores. For instance, circle A indicates that one OCD patient had a LNRT score of 1 and a SRT score of 1, whereas circle B indicates that four patients had a LNRT score of 3 and a SRT score of 3. For each group of patients, the mean Gloden’s inhibition scores obtained in the stroop test are given with their standard deviation, The highly resistant patients (group 4) had significantly lower scores on the stroop test than the non-resistant ones (group 1).

As expected, the cluster analysis that was conducted by combining patients’ LNRT and SRT scores identified four groups of patients (see Figure 1), including a group of 27 highly resistant patients and a group of 16 non-resistant patients. More precisely, the 16 patients of group 1 had low LNRT scores (range 1 to 4) and low SRT scores (range 1 to 3) and were non-resistant patients who responded well to first- and second-line treatments. In contrast, the 27 patients of group 4 had both high LNRT scores (range 7 to 10) and high SRT scores (range 5 to 7). They were highly resistant patients who did not respond well even to high-level treatments. The 12 patients of group 2 had low LNRT scores (range 1 to 4) but higher SRT scores (range 4 to 7) than group 1 patients. They did not respond well to first- and second-line treatments, but their treatment was still evolving and had not reached the highest levels yet, so that no definitive conclusion could be made about their treatment resistance. Finally, the 10 patients of group 3 had high LNRT scores (range 6 to 10) but low SRT scores (range 2 to 4). They were resistant to first and second-line treatments but responded to higher level treatments. Altogether, 75% of the patients (groups 2, 3, and 4) were identified as resistant to first- and second line treatments and/or psychotherapy.

### 3.2. Links between the patients’ clinical variables and scores on cognitive tests

The links between patients’ cognitive abilities and clinical variables were explored using the whole sample of patients involved in the study. The descriptive data for the patients’ executive and working memory tests are given in Table 5.

**Table 5:** Mean values and standard deviations of patients’ scores on executive and working memory tests.

Dependent variable	Patients with OCD	
	*M* ± SD	Range
Verbal fluency score	20.2 ± 5.4	9 to 36
*Stroop test scores*		
Word-reading (card A)	101.5 ± 17.0	70 to 136
Color-naming (card B)	78.7 ± 14.6	45 to 109
Color-word condition (card C)	44.0 ± 13.6	15 to 78
Color-word minus color-naming	−34.7 ± 9.9	−59 to −15
Golden’s inhibition score	−0.2 ± 9.9	−22.2 to 22.8
d2 test score	356 ± 107	160 to 603
d2 error rate (%)	6.0 ± 7.0	0.0 to 37.0
Hayling RT (ms), completion condition	1,050 ± 496	519 to 3,720
Hayling RT (ms), inhibition condition	3,019 ± 1,690	1,002 to 8,752
Number of errors in the inhibition condition	9.8 ± 2.3	4 to 13
Task-switching RT (ms), all trials	1,246 ± 622	534 to 3,479
Task-switching, switching cost (ms)	196 ± 229	−111 to 1,215
Task-switching, incongruency cost (ms)	308 ± 398	−125 to 2,268
Task-switching, error rate (%)	4.2 ± 4.6	0.0 to 22.2
Reading span scores	52.5 ± 14.6	25 to 86
Backward location span scores	111.3 ± 23.4	57 to 157

Sixty-three patients performed the reading span and backward location span tests, whereas 53 of them performed the other tests. Hayling = Hayling sentence completion test, Task-switching = task-switching test, RT = response time.

The results of the task-switching test revealed that both patients’ and controls’ response times to the four different types of trials performed during the test (switch trials, repetition trials, incongruent trials and congruent trials, see methods above) were highly correlated (0.82 <  *r* < 0.96, *p* < 0.001 in all cases). Hence, a single “Task-switching reaction time” corresponding to the average reaction times measured over all types of trials was used in all analyses of the task-switching data (Tables 5, 6), next to the switching cost, incongruency cost and error rate.

**Table 6:** Mean values and standard deviations of patients’ and controls’ scores on executive and working memory tests and results of statistical comparisons.

Dependent Variable	Patients with OCD (*n* = 36)	Controls (*n* = 36)	*t*	df	*p*	*d*
Verbal fluency score	20.6 ± 5.5	20.9 ± 5.5	−0.30	35	0.38	-
*Stroop test scores*						
Word-reading (card A)	105.8 ± 15.9	109.9 ± 17.4	−0.89	35	0.19	-
Color-naming (card B)	81.7 ± 13.8	83.8 ± 15.6	−0.48	35	0.32	-
Color-word condition (card C)	47.1 ± 14.0	50.8 ± 14.4	−0.92	35	0.18	-
Color-word minus color-naming	−34.6 ± 9.7	−33.1 ± 10.0	−0.99	35	0.16	-
Golden’s inhibition score	1.2 ± 10.5	3.4 ± 9.9	−0.91	35	0.19	-
d2 test score	**378 ± 95**	**429 ± 65**	**−2.48**	**35**	0.009*	**−0.63**
d2 error rate (%)	5.2 ± 5.4	4.3 ± 3.4	−0.86	35	0.20	-
Hayling RT (ms), completion	**1,054 ± 529**	**838 ± 168**	**2.35**	**35**	0.012*	**0.55**
Hayling RT (ms), inhibition	**3,080 ± 1,876**	**2,374 ± 778**	**2.29**	**35**	0.014*	**0.49**
Errors in the inhibition condition	10.1 ± 2.6	9.2 ± 2.4	1.33	35	0.10	-
Task-switching RT (ms), all trials	**1,155 ± 485**	**807 ± 277**	**3.89**	**35**	<0.001*	**0.88**
Task-switching, switching cost (ms)	174 ± 162	116 ± 109	1.67	35	0.05	0.42
Task-switching, incongruency cost (ms)	216 ± 208	172 ± 139	0.98	35	0.17	-
Task-switching, error rate (%)	3.3 ± 3.6	3.4 ± 2.4	−0.11	35	0.46	-
Reading span scores	53.5 ± 16.3	56.0 ± 12.7	−0.66	28	0.26	-
Backward location span scores	111.6 ± 25.5	122.8 ± 21.3	−1.94	29	0.032	−0.48

*d* = Cohen’s *d*, *df* = degrees of freedom, Hayling = Hayling sentence completion test, ms = milliseconds, RT = response time, Task-switching = task-switching test. The asterisks (*) indicate the variables for which significant differences were found between patients and controls after correction for multiple comparisons. The numbers in bold indicate significant differences between patients with OCD and their controls.

There was no significant correlation between patients’ insight and Y-BOCS scores (*r* = 0.03 *p* = 0.83), nor between these scores and the patients’ age (−0.04 < *r* < −0.01, *p* > 0.80 in all cases), nor between the patients’ general anxiety level and their age or insight scores (0.00 < *r* < 0.07, *p* > 0.62). There was no significant correlation either between the patients’ scores on executive and working memory tests and the patients’ Y-BOCS (−0.21 < *r* < 0.09, *p* < 0.13 in all cases) or insight score (−0.28 < *r* < 0.28, *p* > 0.05 in all cases). However, the patients’ anxiety level was positively correlated to the severity of their pathology assessed by the Y-BOCS (*r* = 0.51, *p* < 0.001) even after Benjamini and Hochberg’s (62) correction for multiple testing.

### 3.3. Predictors of long-range treatment resistance in patients

As stated above in the methods section, two different but complementary methods were used to analyze treatment resistance data and assess whether the patients’ resistance profile was linked to their scores on executive and working memory tests and/or to clinical variables: multivariate regression models, and comparison between the subsets of highly resistant (group 4) versus non-resistant patients (group 1) identified by the cluster analysis.

#### 3.3.1. Multivariate regression models

Multivariate regression models were built to assess whether each one of the patients’ treatment resistance scores could be predicted by one or several of their scores on cognitive tests and/or clinical variables. Only the 53 patients that performed both the executive and working memory tests were considered for these regression models. In addition, the patients belonging to group 2 identified by the cluster analysis were removed from the analyses because, as stated above, no definitive conclusion could be reached regarding their level of treatment resistance. Hence, only 43 patients were included in the regression analyses.

The regression model that was built to assess on which of the patients’ features LNRT scores depended the most was highly significant [*F*(3,39) = 5.57, *p* < 0.01]. Twenty-five percent of the variance in LNRT scores could be explained by variations in patients’ age, patients’ performance on the Stroop test and patients’ time cost of incongruency in the task-switching test. In accordance with hypothesis 1, high resistance to treatment was associated with lower Golden’s inhibition scores in the Stroop test (b* = −0.35, SE = 0.14, *p* < 0.05), but also with older patients’ age (b* = 0.38, SE = 0.14, p < 0.05) and, unexpectedly, with a lower time cost of incongruency in the task-switching test (b* = −0.31, SE = 0.14, *p* < 0.05). As stated above in the description of the Stroop test in section 2.4, lower, negative Golden’s inhibition scores reflect lower than average inhibition abilities in the Stroop test.

Similarly, the regression model that was built for SRT scores was highly significant [*F*(4,38) = 7.40, *p* < 0.001]. Thirty-eight percent of the variance in SRT scores could be explained by variations in patients’ age, patients’ performance on the Stroop test, patients’ time cost of incongruency in the task-switching test (which were all also predictors for LNRT scores), and response times on completion trials of the Hayling test. In accordance with hypothesis 1, weak response to treatment was associated with lower Golden’s inhibition scores (i.e., lower inhibition performance) in the Stroop test (b* = −0.40, SE = 0.13, *p* < 0.01), but also with older patients’ age (b* = 0.36, SE = 0.13, *p* < 0.01). High SRT scores were also, unexpectedly, again associated with lower time cost of incongruency in the task-switching test (b* = −0.32, SE = 0.13, *p* < 0.05), and with shorter response times on completion trials of the Hayling test (b* = −0.27, SE = 0.13, *p* < 0.05).

#### 3.3.2. Comparison between highly resistant and non-resistant patients

The links between patients’ treatment resistance, clinical variables and cognitive abilities were also explored by comparing the performance of the two extreme groups of patients identified by the joint cluster analysis of LNRT and SRT scores, i.e., the non-resistant patients versus the highly resistant ones (see Figure 1). Compared to the non-resistant patients, this second analysis confirmed that the highly resistant patients were older (42.4 ± 12.4 versus 31.8 ± 10.7 years, *t*(41) = 2.86, *p* < 0.01, *d* = 0.92) and had lower Golden’s inhibition scores on the Stroop test (−2.7 ± 8.2 versus 5.8 ± 12.1, t(33) = −2.44, *p* < 0.05, *d* = 0.82) in accordance with hypothesis 1. In addition, the highly resistant patients displayed higher Y-BOCS scores than the non-resistant patients [28.9 ± 4.6 versus 24.9 ± 4.6, *t*(41) = 2.71, *p* < 0.01, *d* = 0.87].

In summary, the two analyses conducted on patients’ LNRT and SRT scores converged to indicate that long-term treatment resistance was associated with older age at the time of testing, lower inhibition performance in one particular executive test, i.e., the Stroop test, and slightly more severe pathology. Unexpectedly, high treatment resistance as assessed by the LNRT and/or SRT scales was also associated with *quicker* responses on completion trials of the Hayling test and *lower* cost of incongruency in the task-switching test.

### 3.4. Comparison between the cognitive abilities of patients with OCD and matched controls

As explained above, this comparison involved 36 patients versus 36 control participants. In accordance with hypothesis 3, and as shown in Table 6, patients with OCD had significantly lower scores than controls on several of the executive and working memory tests. The patients’ scores on the d2 test of sustained attention were lower than the controls’. The task-switching reaction time was more than 40% longer in patients with OCD than in controls. Similarly, in both conditions of the Hayling test, patients with OCD needed more time to complete the sentences than controls. However, in all three tests (d2, task-switching and Hayling), the error rate did not significantly depend on the pathological status. Other data from the task-switching test revealed that the switching cost was higher in patients with OCD than in controls (Table 6), but the difference did not reach significance after applying Benjamini and Hochberg’s (62) correction for multiple comparisons. The incongruency cost was not significantly different between patients and controls.

For the Stroop test, patients’ inhibition-related scores tended to be lower than the controls’ ones, but the difference was not significant because of the large inter-individual variability of the scores in both groups. Finally, patients’ verbal fluency scores were not significantly different from those of controls.

The working memory tests revealed that the patients’ backward location span scores were lower than those of their matched controls (Table 6), but the difference did not reach significance after correction for multiple comparisons. The reading span scores were not significantly different between the two groups of participants.

## 4. Discussion

### 4.1. Assessment of OCD patients’ treatment resistance

The treatment resistance of patients with OCD was assessed using adaptations of two scales designed by Pallanti and Quercioli (2), i.e., the LNRT scale and the SRT scale. The data show that both scales were well able to capture the inter-individual variability of patients’ treatment resistance. The patients’ LNRT and SRT scores covered the whole range of their respective scales. Even though LNRT and SRT scores were significantly correlated, the cluster analysis performed on the two scores demonstrated that they could be used to unambiguously identify four distinct groups of patients with different resistance patterns, including a group of highly resistant and a group of non-resistant patients. As expected, the LNRT and SRT scales measure two related but separate components of OCD patients’ treatment resistance and combining the two scores accurately represents the patients’ various resistance profiles.

About 75% of all patients (49 out of 65) were resistant to first- and second-line treatments. This proportion is higher than the 40% to 60% of resistant patients reported in the literature (1, 2), but, as pointed out by Howes et al. (8), studies examining hospital populations with chronic illnesses are likely to record higher rates of treatment resistance than studies examining outpatient samples, which include more patients at the onset of illness.

### 4.2. Links between cognitive abilities, treatment resistance, and clinical variables

In accordance with hypothesis 1, higher treatment resistance was associated in patients with OCD with lower inhibition performance on the Stroop test. The patients’ Stroop test scores, and in particular Golden’s inhibition score, were the only scores on cognitive tests that were predictors of both the LNRT and SRT scores, and for which highly resistant patients obtained significantly lower results than non-resistant patients. Since the Stroop test mainly taps the inhibition of prepotent responses (11, 18), this suggests that high treatment-resistance is associated with patients’ inability to block automatic cognitive processes. Interestingly, patients’ treatment resistance was not linked to their performance on the other test that was supposed to tap the prepotent inhibition component, i.e., the inhibition condition of the Hayling test. There was actually no significant correlation between the patients’ performance on the Stroop test and the inhibition condition of the Hayling test. Hence, the inhibition condition of the Hayling test may not measure prepotent inhibition abilities, maybe because the alternation between the completion and the inhibition conditions introduces a task-switching component in the test. Hypothesis 2 was not verified, since in this study the patients’ treatment resistance did not significantly depend on their insight, as already reported by other authors (1, 37). However, as stated in the introduction, at least two experimental studies [(3, 5); see also (28, 35) for reviews] found that OCD patients with poor insight were more resistant to treatments that patients with better insight. Interestingly, the patients with poor insight included in these two experimental studies had very high BABS scores (17 ± 2 and 22 ± 6, respectively) compared to the BABS scores of the samples of patients tested by Alonso et al. (37) (9 ± 5), Eisen et al. (53) (7 ± 5) and in the present study (8 ± 4, maximum score 18). Moreover, in Catapano et al.’s (3) study, almost half of the patients with poor insight had a comorbid schizotypal personality disorder. According to Moritz et al. (63), the presence of positive schizotypal symptoms in OCD patients is associated with a low response to treatment. Therefore, the significant negative relationship between OCD patients’ insight and treatment resistance reported by some authors may result from including a high proportion of OCD patients displaying schizotypal symptoms in the clinical samples. Indeed, according to Beşiroğlu (35), insight may predict differently response to treatment depending on the patients’ various clinical profiles.

As already reported (1, 3, 5, 28, 35), treatment-resistant patients were older and displayed more severe obsessive–compulsive symptoms than other patients. In addition, our data confirmed findings, which found that gender was not a significant predictor of treatment-resistance (1, 5, 27).

The present data may explain why former studies on the relationships between the executive functions and treatment resistance of patients with OCD reported conflicting results (30–33). Indeed, the present data suggest that treatment-resistant OCD only depends on a single component of executive functions, i.e., the inhibition of automatic, prepotent responses, and is not linked to patients’ performance on cognitive tests that tap other executive components. This result confirms the data reported by D’Alcante et al. (4) who found that among patients with OCD, better inhibitory control was predictive of better treatment response. This result also fits well with the anatomical data obtained by Takeda et al. (64), who concluded that the responses to medication of patients with OCD could be predicted by the amount of activation observed in the patients’ prefrontal cortex during Stroop tasks. According to Bannon et al. (65), the patients’ ability to inhibit prepotent responses would be involved in controlling obsessions and compulsions. In particular, low response inhibition abilities in a stop signal task would make patients more prone to compulsions (66). Hence, our data suggest that treatment-resistant patients should be less prone to prevent compulsions than their non-resistant counterparts.

Unexpectedly, higher treatment-resistance was paradoxically associated with some *better* performance on other executive tests than the Stroop test. Indeed, high LNRT and SRT scores were associated with a lower time cost of incongruency in the task-switching test, i.e., better resistance to interference. In addition, high SRT scores were associated with quicker but accurate responses on completion trials of the Hayling test. These data confirm similar observations reported in the literature (4, 34, 67). High treatment resistance in OCD might be associated with better resistance to interference and/or facilitation of automatic responses. Indeed, the treatment-resistant patients who display more severe symptoms and make more perseveration errors are probably less distracted by irrelevant stimuli. In addition, because OCD patients do not adjust their level of cognitive control to changing circumstances and are always “on guard” (14, 66, 68), they may show similar or better scores than control participants on some executive tests when cognitive control is not required for all trials (68). Since in the task-switching test used in the present study, only one-third of the trials involved incongruent stimuli, this may explain why high treatment-resistance and more severe symptoms were associated with a lower time cost of incongruency. Moreover, the lower ability of resistant patients to inhibit automatic cognitive processes should facilitate the recovery of obvious responses in long-term memory during the Hayling test.

As stated above (65, 66), the patients’ ability to control prepotent responses measured by the Stroop task would be particularly involved in controlling compulsions. In contrast, the ability to resist interference might make the individual less vulnerable to recurrent obsessions such as intrusive thoughts, images, and impulses. If treatment-resistant patients with OCD have trouble inhibiting automatic responses but have average or even superior resistance to interference, they should be less prone to prevent compulsions but as prone or more prone to control obsessions than their non-resistant counterparts.

Contrary to what was reported by Kashyap et al. (36), the patients’ insight scores did not depend significantly on either their age or scores on executive and working memory tests. Moreover, no significant correlation was found between OCD severity and the patients’ insight scores, which did not confirm the idea that less insightful patients display more severe symptoms (69, 70). Hence, the presence or absence of significant links between the patients’ clinical variables and cognitive abilities may depend on subtle variations of patients’ clinical characteristics that require further studies. As already reported (15, 16), the patients’ executive and working memory abilities were not significantly linked to OCD severity.

### 4.3. Alterations of executive functions and working memory in OCD patients

According to the literature (15, 40, 71), the performance of patients with OCD on cognitive tests does not depend on their medication status or the type of treatment they receive. Since there was no evidence in the present study that the patients’ treatment status impacted their cognitive abilities, this parameter was not considered in the following discussion.

In accordance with hypothesis 3 and the literature (14–16), patients with OCD exhibited small to moderate deficits across all components of executive functions compared to healthy controls. Indeed, patients’ performance was lower than controls’ one on several executive tests, which collectively called upon each one of the core executive function components defined by Miyake and Friedman (17): inhibition of prepotent response (inhibition condition of the Hayling test), resistance to interference from distracting information (d2 test), working memory updating (completion condition of the Hayling test) and set-shifting (task-switching test). Hence, despite our expectations, tests that specifically tapped the main components of executive functions identified by recent cognitive models did not reveal a more significant alteration of one or the other of these core components in patients with OCD.

More precisely, for the tests tapping the prepotent inhibition component of executive functions, i.e., the Stroop test and the inhibition condition of the Hayling test, patients’ performance was worse than controls’, but the difference only reached significance in the Hayling test (Table 6). Likewise, for the two scores measuring participants’ ability to resist interference, i.e., the d2 score and incongruency cost in the task-switching test, patients’ performance was worse than controls’, but the difference only reached significance for the d2 test. In the task-switching test, response times were much longer for patients than for controls, but the switching cost and error rate were not significantly different between patients and controls. The tests tapping the updating component of executive functions also gave contrasting results, since the verbal fluency scores of patients and controls were not significantly different, whereas, in the completion condition of the Hayling test, patients needed more time than controls to complete sentences. This discrepancy may result from the fact that the verbal fluency test evaluates the efficiency of controlled access to long-term memory, whereas the completion condition of the Hayling test would reflect more automatic recovery of items in long-term memory. Finally, the tests aimed at evaluating working memory revealed that the patients’ location span scores, but not their reading span scores, tended to be lower than those of matched controls, even if the difference did not reach significance. As already reported, patients’ visuospatial working memory was more altered than their verbal working memory (16, 26, 27).

Interestingly, as already pointed out (13, 72), OCD patients took more time than control participants to perform most of the speeded neuropsychological tasks but never made more errors than controls. This suggests that OCD patients could perform executive tasks as well as controls but needed more time to ensure that they made no mistakes. This suggests that part of OCD patients’ deficits in executive functions might be due to their propensity to carefully check what they do (76% of the 66 patients tested in this study displayed checking compulsions). However, further studies are necessary to confirm this hypothesis.

### 4.4. Limitations of the study and conclusion

The first limitation is the final number of patients who were included in the regression analyses involving executive and working memory tests (43), which was relatively low for this type of analysis. Indeed, the adequate sample size for these analyses was estimated to be 47. Despite this limitation, however, both regression analyses gave highly significant models. A second limitation is that to study treatment-resistance over the course of years, we included diverse patients with OCD, some of whom were followed and treated for more than 30 years. Because only the clinical data reported in the patients’ medical files (both in paper and computer versions) were collected, measures of the clinical variables (OCD severity and patients’ insight) at the onset of the disease and treatments were not always available. Hence, the clinical variables entered into the analyses were those available at the time of inclusion in the study, and the data related to clinical variables must be interpreted with this limitation in mind. Another limitation is that because of the large number of different comorbidities displayed by OCD patients, the potential relationships between these comorbidities and patients’ treatment resistance, which may account for some of the inter-individual variability in the data (14), could not be assessed. Moreover, our sample of OCD patients was not that heterogeneous since 75% of the patients were resistant to first- and second-line treatments, and 76% of them displayed checking behaviours. Finally, since the Y-BOCS and anxiety rating scales were not administered to control participants, we cannot exclude that some of them had some low level of obsessive–compulsive symptoms.

Altogether, this study shows that the treatment-resistance of patients with OCD may be quantified over the course of years and treatments using the two scales designed by Pallanti and Quercioli (2). The main result is that among the cognitive abilities of patients with OCD, higher treatment resistance was unambiguously associated with a lower ability to inhibit automatic responses as assessed by the Stroop test. Hence, the Stroop test could be helpful in clinical settings to anticipate the clinical outcome of incoming patients. This study confirms, in addition, that patients with OCD display broad, but relatively moderate alterations of executive and working memory abilities compared to other psychiatric pathologies, and that these cognitive deficits are not linked to the severity of the illness.

More rigorous studies are needed to determine whether the patients’ scores on the Stroop test might accurately predict their treatment outcome. In particular, the specificity and sensitivity of the Stroop test as a cognitive marker of treatment resistance in OCD must be assessed on a much larger number of patients, preferably in a prospective longitudinal and multicentric study. Besides, because using tests that specifically tapped the main components of executive functions did not really clarify the alterations of neuropsychological functions observed in OCD patients, further studies must be undertaken in order to advance our understanding of cognitive functions in OCD. As stated by Kashyap & Abramovitch (14), this may require the incorporation in the neuropsychological assessment of tests that mimic the demands of real-life situations, instead of focusing solely on standard neuropsychological tests.

## Data availability statement

The raw data supporting the conclusions of this article will be made available by the authors, without undue reservation.

## Ethics statement

The studies involving human participants were reviewed and approved by Comité d’éthique du Centre Hospitalier Henri Laborit de Poitiers. Written informed consent to participate in this study was provided by the participants.

## Author contributions

DD, NV, FB, CB, and NJ conceived and designed the study and the experimental material. DD, NV, FB, CB, and IW conducted the experiments and analyzed the data. DD and NV wrote the first draft of the manuscript. FB, CB, and NJ made substantial contributions to the interpretation of the data and wrote sections of the manuscript. AR, IW, BM, and GH-G revised the manuscript critically for important intellectual content. All authors contributed to manuscript revision, read, and approved the submitted version.

## Conflict of interest

The authors declare that the research was conducted in the absence of any commercial or financial relationships that could be construed as a potential conflict of interest.

## Publisher’s note

All claims expressed in this article are solely those of the authors and do not necessarily represent those of their affiliated organizations, or those of the publisher, the editors and the reviewers. Any product that may be evaluated in this article, or claim that may be made by its manufacturer, is not guaranteed or endorsed by the publisher.

## Footnotes

^1^The authors assert that all procedures contributing to this work comply with the ethical standards of the relevant national and institutional committees on human experimentation and the Helsinki Declaration of 1975, as revised in 2008.
